# Delivery Platforms for CRISPR/Cas9 Genome Editing of Glial Cells in the Central Nervous System

**DOI:** 10.3389/fgeed.2021.644319

**Published:** 2021-03-03

**Authors:** Vasco Meneghini, Marco Peviani, Marco Luciani, Giada Zambonini, Angela Gritti

**Affiliations:** ^1^San Raffaele Telethon Institute for Gene Therapy (SR-Tiget), IRCCS San Raffaele Scientific Institute, Milan, Italy; ^2^Department of Biology and Biotechnology “L. Spallanzani”, University of Pavia, Pavia, Italy

**Keywords:** astrocytes, oligodendrocytes, microglia, editing, CRISPR/Cas9 system, adeno-associated viral vectors, lentiviral vectors, nanoparticles

## Abstract

Glial cells (astrocytes, oligodendrocytes, and microglia) are emerging as key players in several physiological and pathological processes of the central nervous system (CNS). Astrocytes and oligodendrocytes are not only supportive cells that release trophic factors or regulate energy metabolism, but they also actively modulate critical neuronal processes and functions in the tripartite synapse. Microglia are defined as CNS-resident cells that provide immune surveillance; however, they also actively contribute to shaping the neuronal microenvironment by scavenging cell debris or regulating synaptogenesis and pruning. Given the many interconnected processes coordinated by glial cells, it is not surprising that both acute and chronic CNS insults not only cause neuronal damage but also trigger complex multifaceted responses, including neuroinflammation, which can critically contribute to the disease progression and worsening of symptoms in several neurodegenerative diseases. Overall, this makes glial cells excellent candidates for targeted therapies to treat CNS disorders. In recent years, the application of gene editing technologies has redefined therapeutic strategies to treat genetic and age-related neurological diseases. In this review, we discuss the advantages and limitations of clustered regularly interspaced short palindromic repeats (CRISPR)/Cas9-based gene editing in the treatment of neurodegenerative disorders, focusing on the development of viral- and nanoparticle-based delivery methods for *in vivo* glial cell targeting.

## Introduction

In the past, there was a “neuron-centric” point of view of neuroscience in which glial cells were mainly relegated to a structural/metabolic supportive role and rarely they were described as key players in the onset of neurodegenerative disorders. This point of view has changed in recent years based on the increased evidence demonstrating that oligodendrocytes and astrocytes (usually defined as neuroglia cells) and microglial cells are key determinants for proper central nervous system (CNS) development and homeostasis. Oligodendrocytes not only are responsible for the generation of myelin sheets, which act as insulators for the transmission of neuronal potentials (Kuhn et al., 2019), but also metabolically support neurons and regulate the action potential firing by secreting ions (e.g., Ca^2+^ and K^+^) (Battefeld et al., 2016), catabolites (e.g., lactate and ATP), neurotrophic factors [e.g., glial cell-derived neurotrophic factor (GDNF), brain-derived neurotrophic factor (BDNF), and insulin-like growth factor 1 (IGF-1)] (Takasaki et al., 2010) and anti-apoptotic agents (e.g., lipocalin-type prostaglandin D synthase) (Taniike et al., 2002). Astrocytes function as the major suppliers of energy substrates (Alberini et al., 2018), secrete and recycle neurotransmitters [e.g., glutamate and gamma aminobutyric acid (GABA)] (Andersen et al., 2019; Durkee and Araque, 2019; Schousboe, 2019), and release axonal guidance and synaptogenic molecules (Fossati et al., 2020), neuromodulators (e.g., d-serine, taurine, l-aspartate, and kynurenic acid) (Durkee and Araque, 2019), and miRNA-loaded exosomes (e.g., miR-26a) (Lafourcade et al., 2016). Thus, astrocytes help to regulate neuronal morphology, synaptic plasticity, and neural transmission. Additionally, astrocytes are involved in the formation and maintenance of the blood–brain barrier (BBB) and in angiogenic processes by releasing the vascular endothelial growth factor, angiopoietin-1/2, and endothelin-1 (Michinaga and Koyama, 2019; Biswas et al., 2020). Lastly, microglial cells are involved in several functions ranging from immune surveillance, synapse sensing and pruning (Paolicelli et al., 2011), neurogenesis (Sierra et al., 2010), and phagocytosis of cellular debris or degenerative neurons.

In neurodegenerative and neurodevelopmental disorders, dysregulation of the neuron–glia and glia–glia networks strongly contributes to neuronal dysfunction and death. The loss of myelin sheets in demyelinating disorders is the result of the dysfunction and death of myelinating oligodendrocytes and impaired/reduced generation of oligodendrocyte progenitor cells (OPCs). This evolves in parallel with neuronal loss and axonal damage produced by altered neuron–oligodendrocyte bidirectional cross talk (Dulamea, 2017).

The loss of normal homeostatic functions and the alteration of the secretome in astrocytes impair synaptic transmission and OPC proliferation/differentiation, leading to abnormal myelination and/or neurodegeneration in Alexander disease (AxD) and hepatic encephalopathy, suggesting that dysfunctional astrocytes can be a primary cause of neurological diseases (Butterworth, 2010; Li et al., 2018; Messing, 2018). Reactive astrocytes release cytokines, components of the extracellular matrix, growth factors, and microRNAs (miRNAs) that modify the local tissue microenvironment, making it either more or less permissive to the regenerative processes (Pekny et al., 2014; Escartin et al., 2019). Reactive astrocytes release pro-inflammatory cytokines, which initially help tissue regeneration by attracting immune cells that clear cellular debris generated by necrotic cells, collapsed microvessels, or destroyed myelin lamellae. Migration of reactive astrocytes in the peri-infarct area scar formation, which limits the spread of inflammation and the progress of neurodegeneration. However, chronic neuroinflammation modifies the microenvironment, ultimately hampering tissue regeneration and contributing to persistent neurological dysfunctions.

Infections or insults to the CNS induce a rapid activation of microglial cells, denoted by a change of cell morphology, surface antigen expression, and the release of cytokines, growth factors, and reactive oxygen species (ROS). Several data support the hypothesis that reactive microglial cells play a pivotal role in neurodegenerative diseases, contributing to the spread of neurodegeneration to other CNS districts and progressing pathological symptoms. In fact, microglial cells shape and remodel the microenvironment by participating in a complex interplay with neurons, other reactive glial cells, and immune cells (monocytes and lymphocytes) (see Colonna and Butovsky, 2017; Xu et al., 2021 for review). Interestingly, the recent application of single-cell analysis platforms (Ajami et al., 2018; Hammond et al., 2019; Miedema et al., 2020) uncovered the highly heterogeneous and multifaceted aspects of microglia responses in neurodegenerative diseases (Masuda et al., 2020). In fact, microglial cells display different cell surface markers and gene expression signatures indicative of a variegate activated phenotype that could be either supportive (with the release of trophic factors like IGF-1 or anti-inflammatory cytokines like IL-10 and IL-4) or neurotoxic [with the upregulation of tumor necrosis factor-α (TNF-α), IL-1β, and NADPH oxidase 2 (NOX2)] depending on the stage of the disease, CNS region, and extent of neuronal demise (Chiu et al., 2008, 2013; Castellani and Schwartz, 2020).

Gene therapy strategies have recently been proposed for the treatment of several neurodegenerative disorders to correct genetic defects and modulate neuroinflammatory pathways in glial cells or to favor astrocyte-to-neuron and astrocyte-to-oligodendrocyte conversions. Engineering of the Cas9 bacterial adaptive immunity response against viruses allowed for the development of methods to generate sequence-specific modifications based on a single-guide RNA (sgRNA) complementary to the target genomic sequence. In the last decade, clustered regularly interspaced short palindromic repeats (CRISPR)-associated Cas9 systems have been applied to *in vivo* genome and epigenome editing in order to disrupt genes, correct mutations, and silence disease-associated factors in different genetic and sporadic diseases affecting the CNS (Cota-Coronado et al., 2019).

Here, we summarize the different applications of CRISPR/Cas9 technologies, focusing on the efficacy and safety of their *in vivo* application for the treatment of neurodegenerative disorders. We discuss the advantages and drawbacks of viral and non-viral gene editing tool delivery, and we propose potential strategies targeting glial cells for the treatment of demyelinating and neurodegenerative disorders.

## Editing Tools to Target CNS Cells

Since the discovery of the CRISPR/Cas9 technology, several molecular engineering efforts have been devoted to the identification and generation of Cas variants, which recognize different protospacer-adjacent motifs (PAMs) to increase the number of genomic targeted loci. This has led to the generation of *Streptococcus pyogenes* Cas9 (SpCas9) variants, which recognize less-restrictive PAMs, allowing precise targeting of almost every genomic locus. In particular, xCas9 and SpG Cas9 enable the recognition of less-restrictive NGN PAMs (Hu et al., 2018; Walton et al., 2020), whereas SpRY Cas9 is able to bind any PAM sequence, with a preferential affinity for NGN and NAN PAMs (Walton et al., 2020). In the perspective of the adeno-associated virus (AAV)-mediated delivery of the CRISPR/Cas9 system, the identification of the SpCas9 natural orthologs *Streptococcus aureus* Cas9 (SaCas9) (Nishimasu et al., 2015) and *Campylobacter jejuni* Cas9 (CjCas9) (Kim et al., 2017a) allows for the generation of a single AAV vector that can carry the expression cassettes for both the nuclease and the sgRNA. This is due to the smaller size of these enzymes being compatible with the ~4.4-kb packaging limit of the AAV genome. As alternative Cas nuclease, the small Class II, Type V Cas12a, which is able to recognize T-rich PAM sequences, can be used for viral delivery (Bin Moon et al., 2018; Jeon et al., 2018; Li et al., 2020).

Safety concerns against the application of CRISPR/Cas9 systems for gene therapy are mainly associated with their potential off-target activity, usually involving genomic loci with up to six mismatched nucleotides compared with the sgRNA, which is complementary to the on-target locus (Martin et al., 2016). Undesired missense or nonsense mutations, small deletions, or translocation events in the genomic regions essential for cell cycle regulation, survival, and metabolism could potentially lead to severe adverse events including tumorigenicity. Strategies are primarily based on the selection of sgRNAs with a low putative off-target frequency, which are designed through the application of algorithms predicting the number and location of mismatches between the sgRNA sequence and the target genome (Manghwar et al., 2020). Additionally, the use of high-fidelity Cas9 proteins engineered to decrease non-specific DNA interactions through the modification of DNA-binding domains could strongly contribute to increase target specificity and minimize Cas9 promiscuity (Kleinstiver et al., 2016; Rees et al., 2017; Vakulskas et al., 2018; Wang et al., 2019; Lee et al., 2020).

**Figure 1:**
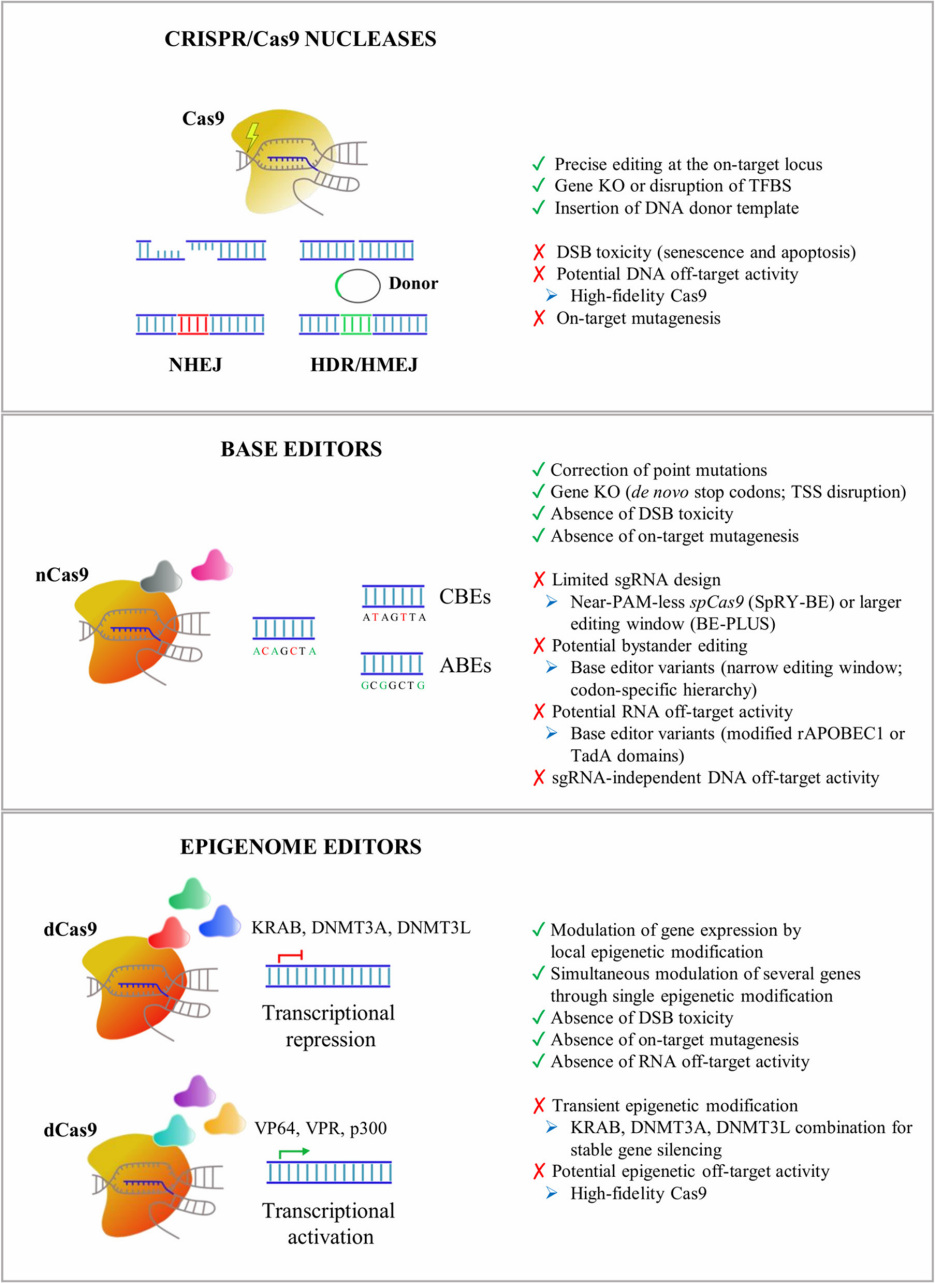
An overview of CRISPR/Cas9 tools applied for *in vitro* and *in vivo* editing of CNS cells. Advantages (green), drawbacks (red), and potential troubleshooting strategies (blue) are listed for Cas9 nucleases, base editors, and epigenome editors. NHEJ, non-homologous end-joining pathway; HDR, homology-directed recombination pathway; HMEJ, homology-mediated end-joining pathway; KO, knock-out; TFBS, transcription factor binding site; DSB, DNA double-strand break; CBEs, cytosine base editors; ABEs, adenine base editors; TSS, transcription start site; KRAB, Kruppel-associated Box; DNMT3A, DNA methyltransferase 3A; DNMT3L, DNA methyltransferase 3L; VP64, four tandem copies of the 16-amino-acid-long transactivation domain (VP16) of the herpes simplex virus (HSV) type 1; VPR, tripartite transactivation complex composed of VP64, NF-κB p65 subunit, and the R transactivator of the Epstein–Barr virus (Rta); p300, histone acetyltransferase p300; CRISPR, clustered regularly interspaced short palindromic repeats; CNS, central nervous system.

Several genome editing tools have been developed for precise and safe human genome engineering leading to the silencing or correction of disease-causing mutations, or the epigenetic regulation of target genes in neural cells (Table 1). Considering the limited editing efficiency in the CNS, the advantages and limitations of each tool have to be carefully evaluated based on the target (genes vs. regulatory regions) and the genomic modification (gene correction vs. silencing of mutated genes vs. the activation of therapeutically relevant proteins) required to achieve the highest therapeutic effect in the treatment of neurodegenerative disorders (Figure 1).

**Table 1:** List of genome editing strategies in CNS cells.

**Editing strategy**	**Targeted gene**	**Delivery platform**	**Animal/cell models**	**Administration route**	**Editing efficiency** **(total nr of edited cells)**	**Edited cells (proportions of different cell types)**					**References**
						**Neurons**	**Astro**	**OLs**	**Müller**	**Microglia**	
NHEJ-mediated gene disruption	*SOD1*	AAV9-SaCas9	SOD1^G93A^ mice (neonatal)	ICV	++	na					Duan et al., 2020
	*Ddit3* and *Sarm1*	AAV2-SpCas9	C57BL/6 WT mice (3/8-week-old)	Intravitreal	~11% (*Ddit3*) ~94% (*Sarm1*)	~11% (*Ddit3*) ~94% (*Sarm1*)	na	na	na	na	Wang et al., 2020a
	*HTT*, GFP and SpCas9 (self-inactivating system)	LV-KamiSpCas9	HD hiPSC-derived neurons and glia		58% (*HTT*) >90% (SpCas9)	+++	+++	na	na	na	Merienne et al., 2017
		LV-CRISPR	Murine striatal neurons		50% (GFP)	100%					
		LV-CRISPR	Murine striatal astrocytes		15% (GFP)		100%				
		LV-KamiSpCas9	Ki140CAG mice (10-week-old)	IP (striatum)	~60% (*HTT*) >90% (SpCas9)	na					
	*GABAα*	IDLV-α2/SpCas9	Murine cortical neurons		++	100%					Ortinski et al., 2017
			Sprague-Dawley rats (adult)	IP (NaC)	+++ in NaC	+++	na	na	na	na	
	APP	AVV9-SaCas9	hiPSC-derived neurons (APP V717I mutation)		+++	100%					Sun et al., 2019
			C57BL/6 WT mice (8-week-old)	IP (hip) ICV	+++	+++	na	na	na	na	
	YFP	CRISPR-Gold (RNP-Cas9)	Thy1-YFP mice (4/8-week-old)	IP (hip)	17–34%	+++	na	na	na	na	Lee et al., 2018
		CRISPR-Gold (RNP-Cpf1)			25–28%	+++	na	na	na	na	
	dTomato	CRISPR-Gold (RNP-Cas9)	Ai9 mice (4/8-week-old)	IP (hip)	10% in hip	10%	50%	na	na	40%	
				IP (striatum)	15% in striatum	10%	50%	na	na	40%	
		CRISPR-Gold (RNP-Cpf1)		IP (hip)	15% in hip	10%	50%	na	na	40%	
				IP (striatum)	15% in striatum	10%	50%	na	na	40%	
	*mGluR5*	CRISPR-Gold (RNP-Cas9)	FMR1 knock-out mice (4/8-week-old)	IP (striatum)	~42% of mGluR+ cells	+++	na	na	na	na	
	dTomato	RNP (4xNLS-Cas9–2xNLS)	dTomato mice (15/20-week-old)	IP (S1)	100 dTomato+ cells/pmol RNP	+++	+	na	na	na	Staahl et al., 2017
				IP (striatum)	150 dTomato+ cells/pmol RNP						
				IP (hip)	100 dTomato+ cells/pmol RNP						
				IP (V1)	~70 dTomato+ cells/pmol RNP						
	eGFP	Cas9 NCs	Tau-eGFP mice (8-week-old)	IP (cer cx)	~50% of eGFP+ cells	+++	na	na	na	na	Park et al., 2019
			Pitx3-eGFP (8-week-old)	IP (midbrain)	~60% of eGFP+ cells						
	*Th1*		C57BL6/J WT mice (8-week-old)	IP (hip)	~70% of Th1+ cells						
	*Bace1*		C57BL6/J WT mice (8-week-old)	IP (midbrain)	~70% of Bace1+ cells						
	*Bace1*		5xFAD mice (6-week-old)	IP (hip)	70% reduction of Bace1 expression	na					
	HIV-1 proviral LTR	AAV9P1	hNSC-derived latGFP1.2 astrocytes/neurons		~5-fold reduction of HIV-1 transcripts	+	+++	na	na	na	Kunze et al., 2018
	*Sox9*	LV.SpCas9-sgRNA	Müller cells isolated from neonatal Sprague–Dawley rats.		80%				100%		Wang et al., 2018a
	*Mertk*	AAV-SaCas9	Sprague–Dawley rats	Intravitreal	+++	na	na	na	+++	na	Koh et al., 2018
Targeted integration (HDR or HMEJ pathway)	Insertion of mCherry sequence at different genomic loci	AAV9-spCas9	murine astrocytes		HDR: ~1%		100%				Yao et al., 2017
					HMEJ: ~2%						
			murine neurons		HDR: ~0.5%	100%					
					HMEJ: ~2%						
			C57BL/6 WT mice (8-week-old)	IP (cortex)	HDR: ~5%	+++	na	na	na	na	
					HMEJ: 52.8% ± 11.3						
			C57BL/6 WT mice (E14.5)	*In utero* electroporation	HDR: ~1%	+++	na	na	na	na	
					HMEJ: 10.0% ± 0.7						
Base editors	*Dnmt1*	v5 AAV-CBE or v5 AAV-ABE	C57BL/6 WT mice (neonatal)	ICV	CBE: 2.5–50%	+++	na	na	na	na	Levy et al., 2020
					ABE: 1.3–43%						
			C57BL/6 WT mice (2-week-old)	IV	CBE: 35–59%	+++	na	+	na	na	
	*Npc1* (c.3182T>C mutation)		Npc1 I1061T (c.3182T>C) mice (neonatal)	ICV	CBE: 0.4% ± 0.51 to 48% ± 8.2	+++	na	na	na	na	
Epigenome editors	pSyn1-iRFP720-GFP	AAV1-PHP.B-dCas9	C57BL/6 WT mice	IV	350–450% increased fluorescence intensity (ventral brain)	+++	na	na	na	na	Lau et al., 2019
	*Scn1a*	AAV-PHP.eB-sgRNA	floxed-dCas9-VPR^VPR/+^/Vgat-Cre^Cre/+^/Scn1a^RX/+^ mice	IV	2/3-fold increased expression of Scn1a (in OB, striatum and neocortex)	+++	na	na	na	na	Yamagata et al., 2020

*In the table are reported the more relevant in vitro and in vivo studies evaluating the editing efficiency upon delivery of Cas9 nucleases (NHEJ, HDR, and HMEJ pathways), base editors (CBEs and ABEs) and epigenome editors in neurons and/or glia cells (Astro, astrocytes; OLs, oligodendrocytes; Müller, müller glia cells; Microglia). Editing efficiency is indicated as downregulation/upregulation of target genes or percentage of edited cells. When quantitative data were not available, a qualitative score (+++, many cells; +, few cells; na, not assessed) based on immunofluorescent analyses is reported in the table. ICV, intracerebroventricular; IP, intraparenchymal; IV, intravenous; NaC, nucleus accumbens; GL, granular layer; ML, molecular layer; Hip, hippocampus; V1, visual cortex; S1, somatosensory cortex; OB, olfactory bulb; LV, lentiviral vector; AAV, adeno-associated vector*.

### CRISPR/Cas9 Nucleases

CRISPR/Cas9 nucleases enable precise genome editing by inducing DNA double-strand breaks (DSBs) at selected genomic loci. These are then repaired by the more accurate (but less active) homology-directed recombination (HDR) pathway or by the more active (but error-prone) non-homologous end-joining (NHEJ) pathway.

Among the repair mechanisms adopted by the cell upon DSBs, the more active error-prone NHEJ pathway induces insertion or deletion events (InDels) of various lengths that can potentially lead to frame-shift mutations in the coding sequence of the target genes resulting in premature stop codons and consequently gene knock-out (KO). Several preclinical studies have demonstrated the advantages of CRISPR/Cas9-mediated gene disruption for the treatment of both autosomal dominant genetic and sporadic neurodegenerative disorders (e.g., Merienne et al., 2017; Park et al., 2019; Sun et al., 2019). A single-dose of CRISPR/Cas9-based treatment might be a more effective and safer approach to downregulate the expression of target genes when compared with DNA antisense oligonucleotides (ASO), which have recently been proposed in preclinical studies for the treatment of inherited disorders affecting astrocytes and oligodendrocytes, like AxD and Pelizaeus-Merzbacher disease (PMD) (Hagemann et al., 2018; Elitt et al., 2020). Although effective in animal models, the development of ASO-based approaches could be time-consuming and costly, and their clinical application poses safety concerns due to the need for chronic administration of relatively high doses to produce a therapeutically relevant level of knock-down of the target protein (Walters et al., 2015). The successful NHEJ-mediated disruption of target sequences has recently been described in glial cells. AAV delivery of two sgRNAs targeting the LTR-containing region of HIV-1 proviruses resulted in a reduced proviral reactivation in an *in vitro* model for HIV-1 transcriptional latency in astrocytes (Kunze et al., 2018). *In vitro*, lentiviral vector (LV) delivery of the CRISPR/Cas9 system strongly downregulated the expression of the *Sox9* gene in primary retina Müller cells (Wang et al., 2018a). *In vivo*, the successful knock-down of *Mertk* (Mer Receptor Tyrosine Kinase) has been achieved in Müller glia cells after an intravitreal injection of a SaCas9-sgRNA AAV vector in the eyes of P7–P10 rat pups (Koh et al., 2018).

The HDR pathway directs precise recombination events that can be exploited for the accurate insertion of a donor template. Recently, homology-mediated end-joining (HMEJ)-based strategies have been proposed for the efficient and precise *in vivo* targeted integration into the visual cortex, making the design of homology-dependent gene correction strategies in post-mitotic neurons and astrocytes practicable (Yao et al., 2017). HMEJ-based strategies could be applied for the gene correction of several recessive genetic neurodegenerative disorders. These approaches are particularly relevant in diseases not amenable to gene addition due to the fine-tuned regulation required to achieve a therapeutic effect, i.e., the correction of PMD-causative point mutations identified in patients affected by *PLP*1 haploinsufficiency.

A major safety concern in the application of Cas9 nucleases is the DSB-induced toxicity leading to senescence and apoptosis in the target cells (Cromer et al., 2018). Although the impact of CRISPR/Cas9-induced DSB toxicity in neurons and glial cells is still unclear, the application of high-fidelity Cas9 nucleases and the selection of sgRNA with low predicted homology for off-target loci might reduce DSB events (Schiroli et al., 2019). Despite the continuous efforts in improving Cas9 specificity, attention must be given to potential on-target mutagenesis (e.g., large deletions/inversions and complex genomic rearrangements) that not only disrupts the target genomic locus but also could potentially elicit long-range transcriptional misregulation of oncogenes (Kosicki et al., 2018).

### Base Editors

Base editors have recently been generated by fusing catalytically inactive dead Cas9 (dCas9) or nickase Cas9 (a mutated nuclease generating a nick only in one strand) with enzymes able to chemically convert single nucleic bases.

Cytosine base editors (CBEs) contain a cytidine deaminase domain, which catalyzes the hydrolytic deamination of cytosine to uracil within the single-stranded R-loop generated by Cas9 in the sgRNA-recognized DNA sequence (Komor et al., 2016, 2018). Third-generation CBEs (BE3) have been engineered to improve the editing efficiency by the addition of an uracil glycosylase inhibitor (UGI) that inhibits the activity of uracil glycosylases, responsible for the excision of uracil bases and the generation of apurinic/apyrimidinic sites, which promote base scrambling and InDel formation. Additionally, the use of nickase Cas9 (nCas9) further increases the base editing rate by forcing the DNA repair machinery to use uracil in the repair of the nicked template and favors the final C–G to T–A base pair conversion (Komor et al., 2016).

Conversely, adenine base editors (ABEs) are able to convert A–T base pairs into G–C base pairs, thanks to a heterodimer composed of a wild-type (WT) non-catalytic monomer from the *Escherichia coli* tRNA adenosine deaminase enzyme (TadA), which contributes to DNA binding, together with an evolved TadA^*^ monomer, which deaminates the exocyclic amine of adenine, thus generating an inosine intermediate, which exhibits a base pairing preference for guanosine (Gaudelli et al., 2017). The nick introduced by nCas9 directs the DNA repair machinery to incorporate a cytosine opposite to the inosine and subsequently to install a guanosine on the deaminated strand (Gaudelli et al., 2017). Notably, inosine is also a substrate for excision by cellular glycosylases, which can generate apurinic/apyrimidinic sites, although InDel frequencies are typically below 1% in treated cells (Koblan et al., 2018; Ryu et al., 2018; Yeh et al., 2018) and mice (Ryu et al., 2018). Recently, ABEs have also been described for their ability to convert cytosine to guanine or thymine in a narrow editing window (positions 5–7) and in the context of a confined TC^*^N sequence. This occurs independently of adenine conversions (Kim et al., 2019), thus broadening their applications for high-specificity base editing.

Almost 58% of the genetic variants in human diseases are associated with point mutations, including mendelian-segregating genes and single-nucleotide polymorphisms (SNPs) associated with genetic risk factors in neurodegenerative disorders (Bertram and Tanzi, 2005). Of these, nearly 50% of the most common pathogenic point mutations could be reversed by the deaminase activity of CBEs and ABEs (Rees and Liu, 2018). Additionally, CBEs are an alternative to Cas9 nucleases to induce the knock-down of mutated genes, being able to generate *de novo* stop codons without inducing DSBs, thus circumventing the risks associated with DSB toxicity. Similarly, silencing of a target gene can be induced with an ABE-mediated start codon mutation (from ATG to GTG or ACG), as demonstrated by the knock-down of the murine programmed cell death protein 1 (PD-1) gene in Neuro-2a cells and mouse pups (Wang et al., 2020b). In human astroglial-like cell lines, base editors have recently been applied to reproduce the cancerogenic heterozygous mutation IDH1^R132H/WT^, demonstrating the feasibility of base conversion even in glial cells (Wei et al., 2018).

Since base editors rely on the accessibility of deaminase enzymes to the adenines or cytosines within the R-loop, the editing window is restricted (e.g., positions 4–8 for SpCas9), resulting in the limited identification of targetable genomic loci. This drawback could be circumvented by using base editing machineries with a broader editing window (e.g., positions 4–14 for BE-PLUS) (Jiang et al., 2018) or recognizing less-restricted PAMs (e.g., near-PAM less SpRY-BEs or SpG-BEs) (Walton et al., 2020) to improve sgRNA design. It is important to note that multiple editable adenines or cytosines could exist within or nearby the editing window, leading to the undesired conversion of non-target nucleotides (bystander editing). To minimize bystander editing, a careful design of sgRNAs can be combined with the application of base editor variants with altered activity windows. For example, mutations in the rAPOBEC1 domain of CBE (YE1-BE3, YE2-BE3, and YEE-BE3) resulted in a narrow editing window, enabling the selective conversion of a target cytosine (Kim et al., 2017b). Alternatively, the evolution of the human APOBEC3A domain gave rise to the eA3A-BE3 editor that preferentially deaminates cytidines according to a specific TCR > TCY > VCN hierarchy (Gehrke et al., 2018).

Two recent whole-genome sequencing analyses in edited murine blastomeres (Zuo et al., 2019) and rice plants (Jin et al., 2019) revealed a high amount of sgRNA-independent off-target single-nucleotide changes in highly transcribed regions. These off-target events were significantly higher by using BE3 as compared with ABEs, and they were due probably to R-loop formation during the transcription process that increased the accessibility of the cytidine deaminase domain to unrelated genomic loci (Jin et al., 2019).

In addition to DNA off-targets, RNA off-target activity has recently been described. Recent studies on the transcriptome of CBE-edited mammalian cells showed the presence of C-to-U modifications, preferentially in the ACW sequence motif (W = A or U), which are not caused by corresponding mutations introduced by DNA editing (Grunewald et al., 2019). A similar RNA off-target activity has also been detected for ABEs (Grunewald et al., 2019; Rees et al., 2019; Zhou et al., 2019). To circumvent RNA editing, BEs have been engineered by introducing R33A or R33A/K34A modifications into the rAPOBEC1 domain (Grunewald et al., 2019) or by adding bulky or hydrophobic amino acids in the TadA domains (Rees et al., 2019; Zhou et al., 2019). Both these modifications generate a steric clash with RNA molecules leading to a reduced C-to-U conversion, while maintaining similar DNA on-target efficiency.

### Epigenome Editors

An intriguing development of the Cas9 technology is the generation of tools based on the combination of transcriptional and epigenetic modulators with catalytically inactive dCas9 to modulate the expression of specific target genes. The first evidence of these mechanisms came from the observation that binding dCas9 to a region spanning from −55 to +20 bp in the promoter hampered the recruitment of transcription factors (TFs) and RNA polymerase II and induced target gene silencing (Qi et al., 2013). The fusion of dCas9 with Kruppel-associated Box (KRAB), which in turn recruits the KRAB-box-associated protein-1 (KAP-1) and epigenetic readers [e.g., heterochromatin protein 1 (HP1)], further enhanced the repressive potential of dCas9 (Gilbert et al., 2013). From these preliminary evidences, the KRAB-based system was improved by using a catalytic domain of the eukaryotic DNA methyl transferases (DNMT3A and DNMT3L) to decorate regulatory regions with repressive methylation marks and recruit other repressive proteins (e.g., polycomb complex) that induce a strong and stable gene silencing (Amabile et al., 2016; Liu et al., 2016; Stepper et al., 2017). Indeed, methylation of CpG islands, often located within the promoter region, can result in epigenetic silencing (Amabile et al., 2016; Liu et al., 2016).

In parallel, CRISPR/Cas9 activator tools have been generated by fusing the dCas9 protein with strong transcriptional activators. The 16-amino-acid-long transactivation domain (VP16) is a TF of herpes simplex virus (HSV) type 1, which is involved in the activation of the viral immediate–early genes. It binds the host cell factor (HCF) nuclear proteins and the octamer transcription factor-1 (Oct-1) generating a protein complex able to activate genes through interactions between the transcriptional activation domain and several other TFs (Hirai et al., 2010). Increased epigenetic activity has been achieved by fusing the dCas9 with four tandem V16 copies leading to the generation of the epigenetic activator dCas9–VP64 (Maeder et al., 2013; Mali et al., 2013; Perez-Pinera et al., 2013). In parallel, a stronger epigenome editor has been generated by using a tripartite (VPR) transactivation complex composed of VP64, NF-κB p65 subunit, and Rta (the R transactivator of the Epstein–Barr virus) (Chavez et al., 2015). Recently, the SunTag complex has been generated by fusing dCas9 with a protein scaffold containing repeat array peptides able to recruit multiple copies of an antibody linked to different effector proteins (Tanenbaum et al., 2014). In alternative, dCas9 systems based on epigenetic proteins that promote the demethylation of DNA (i.e., Tet1) (Liu et al., 2016) and histones (i.e., LSD1, a histone demethylase that removes H3K4me2) (Kearns et al., 2015) or promote histone H3K27 acetylation (i.e., p300 catalytic domain) (Hilton et al., 2015) can be applied to activate target genes. A complete list of Cas9-based tools for epigenome editing has been reviewed in (Liu and Jaenisch, 2019).

Epigenome editing tools have been applied to target both promoters and enhancers in order to highly activate or repress a specific gene. For promoter targeting, it has usually been observed that gene activation and gene repression require different sgRNA positions with respect to the transcription start site (TSS). Transcriptional activators are usually directed upstream of the TSS (from −1,000 to +1 bp) with the highest levels of activity observed by targeting the region from −200 to +1 bp in the promoter (Konermann et al., 2015). On the contrary, the dCas9–KRAB system usually provides stronger and higher specific suppression by using sgRNA targeting regions located 50–100 bp downstream of the TSS (Gilbert et al., 2013). An alternative strategy is based on the targeting of enhancers to modulate transcriptional activation/repression in a cell-specific manner. In a study by Gersbach et al., they demonstrated the ability to target proximal and distal enhancers of specific genes and boosting transcriptional activation by using a dCas9–p300 complex (Hilton et al., 2015). They also demonstrated, in parallel, the possibility to silence an individual enhancer with a high degree of specificity by using a dCas9–KRAB system capable of inducing local epigenetic modifications (Thakore et al., 2015). Considering that an enhancer could drive the expression of multiple genes, epigenome editing of these regulatory regions could result in the simultaneous modulation of several genes through a single epigenetic modification; however, this could be advantageous or disadvantageous, depending on the genomic context and target genes (Hilton et al., 2015; Polstein et al., 2015).

Epigenetic alterations have been observed in several brain pathologies (Landgrave-Gomez et al., 2015), making the epigenome editing an intriguing therapeutic strategy to regulate gene expression in complex neuropsychiatric disorders or activate genes involved in haplodeficient diseases. Two *in vivo* studies demonstrated that targeting H3K9 acetylation or methylation of the *FosB* gene in the nucleus accumbens can influence behavioral susceptibility to cocaine addiction or the response to social stress in mice (Heller et al., 2014; Hamilton et al., 2018). Additionally, the reactivation of the *FMR1* gene through dCas9–Tet1-induced demethylation of cytosines in CGG repeats (Liu et al., 2018) or dCas9–VP192-induced transcriptional activation of the *FMR1* promoter (Haenfler et al., 2018) restored the spontaneous hyperactivity in neurons derived from human induced pluripotent stem cells (hiPSCs) of patients affected by Fragile X syndrome (FXS).

The long-term efficiency of epigenome editing approaches is strictly correlated with the stability of the newly generated epigenetic changes, which could be re-converted in the absence of permanently expressing editing tools. The dynamic mechanisms operating to ensure the epigenetic inheritance of DNA methylation, the binding of DNA- and chromatin-associated factors, and the histone modifications are not yet completely understood (Probst et al., 2009). In the perspective for the application of epigenome modifiers in glial cells, it is relevant to understand whether epigenetic changes, once established, are stable in daughter cells or maintained during events characterized by relevant modifications of the transcriptional and epigenetic landscapes, such as polarization in microglia, astrogliosis, or maturation of the oligodendroglial repertoire. The combination of DNA methylation with H3K9me3 modification improved the stability of *FMR1* activation for up to 4 weeks in FXR hiPSCs, although it is not clear if it is reproducible in more committed cells (Liu et al., 2016). The permanent epigenetic modification of target genes is therapeutically relevant for the treatment of genetic disorders affecting the CNS; however, a transient activation or repression of genes involved in microglia polarization or astrogliosis could be relevant to temporally boost anti-inflammatory and pro-neurogenic effects without compromising the long-term functionality of these cells.

Beside the sgRNA-dependent off-target effects that could be prevented by using high-fidelity dCas9, the permanent expression of an epigenome editor may produce non-specific epigenetic modifications resulting in long-range epigenetic changes that could influence the expression of other non-target genes (Groner et al., 2010). Galonska et al. (2018) observed genome-wide gRNA-independent off-target activity, by tracking the dCas9–DNMT3A footprint in a murine embryonic stem cell line and in two somatic human cell lines. A combination of KRAB, DNMT3A, and DNMT3L has recently been applied to provide stable and highly specific DNA methylation at the target locus, which is increased in the presence of CpG-free boundaries flanking the targeted CpG islands that could prevent the spreading of the epigenetic modifications to neighboring genes and reduce off-target effects (Amabile et al., 2016).

## Viral Delivery of CRISPR/Cas9 Systems

### Strategies to Improve the Transduction of Glia Cells by Adeno-Associated Vectors

AAVs have been extensively used in several rodent and non-human primate (NHP) preclinical studies to deliver therapeutic proteins, miRNAs, and CRISPR/Cas9 systems for the treatment of neurodegenerative disorders (Deverman et al., 2018). The relative safety of AAV gene therapy has been demonstrated in more than 200 pediatric and adult patients affected by several different CNS disorders (Svetkey et al., 1987; Uchitel et al., 2020). The promoter driving the transgene expression, the AAV capsid, and the route of administration are key determinants in defining the homogeneity of AAV transduction across different CNS regions, the cell tropism, and the cell-type specificity of transgene expression in astrocytes and oligodendrocytes (Table 2). In contrast, targeting microglial cells with AAV remains challenging, despite some recent promising results (Rosario et al., 2016; Grace et al., 2018; Maes et al., 2019) (Table 2).

**Table 2:** List of pre-clinical studies for AAV- and LV-mediated targeting of glial cells in the central nervous system.

**Viral vector**	**Cell target**	**Route of administration**	**Animal/cell models**	**Transduction efficiency**	**References**
AAV6-gfaABC_1_D-EGFP-miR124T	Astrocytes		Primary cortical cells from embryonic day 18 rat pups.	gfaABC_1_D promoter was not selective for astrocytes *in vitro* (66.9 ± 11.1% astrocytes and 33.1% neurons).	Taschenberger et al., 2017
		IS	Young adult female Wistar rats	miR-124 detargeted transgene neuronal expression *in vitro* and *in vivo*, but lower transduction efficiency has been reported.	
AAVDJ8-GFAP-mCherry			Primary cortical neurons and mixed glia from P0 neonatal C57BL/6 mice	Astrocytes: 83.2% ± 6.5 mCherry+/GFAP+ cells (primary cortical cultures).	Hammond et al., 2017
		ICV	P0 neonatal C57BL/6 mice.	Astrocytes: 80.3 ± 6.3 mCherry+/S100β+ cells (3 weeks post-injection).	
AAV2/5-CBA-EGFP		ICV	Neonatal (p0, p2, and p3) B6C3F1/Tac mice	Time- and serotype-dependent distribution: AAV2/8 and AAV2/9 displayed the widest tissue distribution.	Chakrabarty et al., 2013
scAAV9-CBA-GFP		IV	Neonatal and 70-day-old C57Bl/6 mice	Neuronal and astrocytes transduction in neonates. 90% of astrocytes transduction in adult spinal cord.	Foust et al., 2009
rAAV-dsCAG-GFP (ShH19, ShH13, and L1-12 capsids)	Astrocytes, Müller cells	IS, subretinal area	Adult Fischer rats, adult Sprague Dawley rats	ShH19 and L1-12 transduced 5.5-fold (14.9 ± 3.0%) and 3.3-fold (9.0 ± 3.0%) higher numbers of astrocytes compared to AAV2	Koerber et al., 2009
rAAV2-RSV-βgal rAAV4-RSV-βgal rAAV5-RSV-βgal	Astrocytes, Neurons	IS, ICV	6/8-week-old C57BLy6 mice	Higher transduction efficiency of rAAV4 and rAAV5 in the striatum (15 weeks post-injection). Higher transduction efficiency of rAAV5 in the ventricle (3 and 15 weeks post-injection).	Davidson et al., 2000
AAV4-RSV-βgal		IV, SVZ	Newborn and young adult C57BL/6 mice	Low number of NeuN+ cells in the OB (IV). High GFAP+ cells in the OB (SVZ).	Liu et al., 2005
AAV-PHP.eB AAV-PHP.S		IV	6/8-week-old C57BL/6J mice	AAV-PHP.eB transduced 69% of cortical and 55% of striatal neurons. AAV-PHP.S transduced 82% of dorsal root ganglion neurons, as well as cardiac and enteric neurons	Chan et al., 2017
AAV2-CMV-GFP (hu.32, hu.37, hu.11, pi.2, hu.48R3, and rh.8 capsids)	Astrocytes, Oligodendrocytes	ICV (neonatal), IP (adult)	Neonatal and adult C3H/HeOuJ mice	Higher transduction efficiency of hu.11 (4.54% ± 2.19 GFP positive area). hu.32 and hu.48R3 led to GFP expression in astrocytes.	Cearley et al., 2008
bdLV.GALC.GFP		EC	FVB/Twitcher mice	3% of astrocytes (GFAP+), 8% of oligodendrocytes (APC+), and <2% macrophages/microglia (CD68+ and Iba1+ cells).	Lattanzi et al., 2014
LV.hARSA LV.GFP	Astrocytes, Oligodendrocytes, Neurons	IP	Macaca fascicularis	22.3 ± 5.7% of astrocytes (GFAP+), and 24.4 ± 10.6% (CNPase+), 50.5 ± 5.7% of neurons (NeuN+)	Meneghini et al., 2016
AAVrh.10-CAG-cuARSA	Oligodentrocytes	IS, IP	8-month-old MLD mice	>90% neurons, 21.4% ± 1.1 oligodendrocytes in the corpus callosum and the internal capsules.	Piguet et al., 2012
AAV9EU-CBA-mCherry AAV9AU-CBA-mCherry		IC	Sprague-Dawley rats	Neurons (14.2% ± 3.6), oligodendrocytes (79.9% ± 4.6) for AAV9EU-CBA-mCherry. Neurons (89.8 ± 3.9%), oligodendrocytes (2.1 ± 0.8%) for AAV9AU-CBA-mCherry	Powell et al., 2020
Olig001-CBh-GFP			Mixed glial cultures from p3 C57BL/6J pups.	9-fold higher transduction efficiency in glial cells with respect to AAV8.	Powell et al., 2016
		IS	Adult female C57Bl/6 mice.	>95 striatal oligodendrocytes.	
LV.CNP.IRES.EGFP		ICV, IP	Neonatal C57BL/6 mice	20.3 ± 2.56% of oligodendrocytes in different CNS regions.	Kagiava et al., 2014
rAAV5-F4/80-RFP vector	Microglia		Primary rat microglia cultures from p1-p2 pups.	Efficient transgene expression in microglia.	Cucchiarini et al., 2003
		IS	Sprague–Dawley rats.	Selective microglia tropism.	
F4/80-GFP and CD68-GFP expression cassettes in AAV6-TM6 (Y731F/Y705F/T492V mutated capsid)			Primary neuroglia and microglia cultures from neonatal mice.	>95% transduction of primary microglial cells.	Rosario et al., 2016
		ICV, IP	Neonatal (ICV) and 2-month-old (IP) B6/C3H mice.	Selective microglia tropism.	
rAAV2/6-based recombinant genomes-CMV-eGFP/RFP			Mixed neonatal cortical glia cultures from p3-p4 and adult C57/BL6 mice.	98 and 99% of RFP+ cells in newborn and adult microglia (rAAV2-CMV-eGFP). 80-fold higher transgene expression (rAAV6-CMV-eGFP).	Su et al., 2016
LV.PGK.GFP LV.PGK.GFP.miR 9.T		IS	Sprague–Dawley rats	75% of GFP+ microglia (Iba1+) and 1% of GFP+ striatal neurons (DARPP-32+).	Akerblom et al., 2013
LV.CMV.Twitch-2B.miR 9.T		IP	2/6-month-old C57BL/6 mice	36.58% of transduced microglia (Iba1+).	Brawek et al., 2017
rAAV1-CMV-IE-GFP	Microglia, Astrocytes, Oligodendrocytes	IS	Adult C3H/HeJ mice	Astrocytes (751 ± 122 cells), oligodendrocytes (164 ± 24 cells), and microglia (101 ± 35 cells).	Wang et al., 2003

*In the table are reported in vitro and in vivo data on AAV and LV transduction efficiencies in glial cells. IS, Intrastriatal injection; ICV, Intracerebroventricular injection; IP, Intraparenchymal injection; IC, Intracranical injection; IV, Intravenous injection; EC, External Capsule injection; gfaABC_1_D, minimal GFAP promoter; CBA, chimeric CMV–chicken ß–actin promoter; RSV, Rous Sarcoma Virus Long Terminal Repeat promoter; CMV, Cytomegalovirus promoter; CAG, composite of the CMV early enhancer and chicken beta-actin promoter; CBh, 800-bp hybrid form of the CBA promoter; F4/80 and CD68, microglia-specific promoters; PGK, Phosphoglycerate Kinase promoter*.

Cell-type specific promoters can be used to enhance and restrict transgene expression in astrocytes, oligodendrocytes, or microglia. The presence of a 2.2-kb human *GFAP* promoter (gfa2) is sufficient to preferentially drive transgene expression in astrocytes; however, its large size hampers packaging it into the AAV genome (Lee et al., 2008). By dissecting the regulatory regions of the gfa2 promoter, the minimal 681 bp gfaABC(1)D promoter has recently been identified and characterized, showing a 2-fold higher activity and a widespread expression pattern across different brain areas (Lee et al., 2008). The gfaABC(1)D(B3) variant containing three copies of the B enhancer element improved 6-fold the transgene expression in astrocytes when compared with the gfaABC(1)D promoter (Humbel et al., 2020). On the contrary, the gfaABC(1)(mC(1.1))D promoter variant showed restricted transgene expression in the astrocytes of the dorsal and caudal cortices, hippocampus, and caudal vermis of the cerebellum (Lee et al., 2008).

Truncated versions of the oligodendroglial-specific recombinant *Mag* promoter (2.2, 1.5, and 0.3 kb) have been successfully tested in neonates and adult mice, resulting in long-term oligodendrocyte-specific transgene expression upon an intraparenchymal AAV injection. Interestingly, the truncated CBA hybrid (CBh) promoter increased gene expression in striatal oligodendrocytes, and the insertion of a six-glutamate peptide immediately after the VP2 start residue in AAV9 capsid shifted CBA-driven expression from neurons to oligodendrocytes (Powell et al., 2020). In addition, myeloid lineage-specific promoters, like F4/80 and CD68, can be used for transgene expression in microglia and in monocyte-derived infiltrating macrophages (Cucchiarini et al., 2003; Rosario et al., 2016).

While lineage- and cell-specific promoters improve/restrict transgene expression in selected cell populations, they are usually characterized by a low but still significant off-target expression in other cell types. Therefore, miRNA de-targeting strategies based on the inclusion of sequences complementary to endogenous miRNAs that are selectively expressed in off-target cell populations could be applied to further increase cell specificity. The incorporation of three miRNA target sequences complementary to the neuronal-specific miRNA-124 can de-target transgene expression in neurons, thus further restricting transgene expression in glial cells (Merienne et al., 2017; Taschenberger et al., 2017; Humbel et al., 2020). Similarly, the introduction of tandem repeats of the miRNA-9 binding sites in the 3′UTR of the transgene could increase microglia specificity, due to the fact that miRNA-9 is expressed in all other neural cell types except murine microglia (Akerblom et al., 2013). To decrease the off-target expression in peripheral organs, while using AAV systemic delivery, sequences complementary to miRNA-122, expressed in the liver, and to miRNA-1, expressed in skeletal muscles, could be inserted downstream to the transgene coding sequence with negligible effects on CNS expression (Xie et al., 2011). Finally, to selectively degrade the transgene mRNA in antigen-presenting cells, miRNA-142-3p target sequences can be incorporated to potentially reduce Cas9 immunogenicity (Majowicz et al., 2013). The approach based on miRNA de-targeting is particularly intriguing considering that the short length of miRNA sequences allows their multiplexing for greater refinement of the post-translational regulation of transgene expression.

The extent of biodistribution in the CNS is influenced by multiple factors of the AAV capsid, including the interactions with receptors and the anterograde and retrograde axonal transports. Despite the relatively restricted biodistribution, AAV2 has been one of the most well-characterized serotypes used in humans for neurological applications due to the fact that it can ensure long-term transgene expression in the CNS (Worgall et al., 2008; Rafii et al., 2014, 2018; Warren Olanow et al., 2015; Niethammer et al., 2017; Chu et al., 2020). Among the natural AAV serotypes, some extent of astrocyte transduction has been reported after intracerebroventricular (ICV) injection of AAV4 and AAV5 (Davidson et al., 2000; Liu et al., 2005). AAV4 only transduces astrocytes within the subventricular zone, whereas AAV5 transduction of astrocytes was highly variable, possibly as a consequence of differences in vector production and promoter usage (Davidson et al., 2000; Liu et al., 2005). Recently, AAV9 showed a higher transduction efficiency of astroglial populations, even when injected intravenously (Foust et al., 2009), whereas a good tropism for oligodendrocytes within white matter tracts has been observed for AAV1 (Wang et al., 2003), and for hu.32, hu.11, pi.2, hu.48R3, and rh.8 serotypes (Cearley et al., 2008). Although the clinical benefits are still under evaluation, AAVrh.10 has recently been applied to deliver the lysosomal arylsulfatase A enzyme in a clinical trial for the treatment of demyelinating metachromatic leukodystrophy (NCT01801709) (Zerah et al., 2015), after promising results were achieved in mice (Piguet et al., 2012) and NHPs (Rosenberg et al., 2014).

To increase cell selectivity and tropism, AAV hybrid serotypes have been generated by viral engineering through the integration of the genome containing (*cis*-acting) inverted terminal repeats (ITRs) of a CNS-permissive serotype with the capsid genes of other serotypes. The hybrid serotype AAV2DJ8 displayed a high *in vitro* transduction efficiency in murine astrocytes and a wide rostro-caudal distribution in various brain regions with prevalent targeting of astroglial populations upon ICV injection in neonatal mice (Hammond et al., 2017). Of note, astrocyte specificity of transgene expression was further increased by using the gfaABC(1)D promoter (Hammond et al., 2017). Additionally, Powell et al. (2016) developed a chimeric mixture of AAV1, 2, 6, 8, and 9 (named Olig001), with a 95% tropism for striatal oligodendrocytes and lower transduction of peripheral organs. On the other hand, to increase the tropism for microglia, Su et al. (2016) tested several AAV2, 5, 6, 8, and 9 pseudotyped AAVs, demonstrating that AAV2/6 displayed a higher efficiency in transducing primary murine microglial cultures. *In vivo*, an AAV2/9 containing a DREADD (Designer Receptor Exclusively Activated by a Designer Drug) system driven by the CD68 promoter was able to induce transgene expression exclusively in Iba1-positive cells upon intrathecal administration (Grace et al., 2016).

To further increase AAV biodistribution in CNS tissues and transduction efficiency in glial populations, AAV capsids have been engineered by directed evolution or structural mutagenesis. The group of David V. Schaffer has produced a panel of highly diverse (>10^7^ members each) AAV libraries generated by random mutagenesis, DNA shuffling, AAV peptide display, and a new semi-random loop replacement method. The AAV libraries were then selected via multiple evolutionary cycles, or genetic diversification, on primary human astrocytes (Koerber et al., 2009). The most notable AAV2 variants were ShH19 and L1-12, which transduced both human and rat astrocytes *in vitro* with an efficiency of up to 15-fold higher than their parent serotypes (Koerber et al., 2009). These AAV variants also exhibited an enhanced infection of astrocytes (up to 16% of the total transduced cell population) upon injection into the rat striatum (Koerber et al., 2009). A small-scale library of chimeric AAV capsids derived from five natural AAV serotypes (AAV1, 2, 6, 8, and 9) has recently been generated and tested in human astrocytes and hiPSC-derived organoids by integrating specific 7- to 9-amino-acid-long peptides (Kunze et al., 2018). The variant (AAV9P1) that most efficiently transduced the astroglial population contained a peptide (P1) enriched with an Arg-Gly-Asp (RGD) motif that is known to mediate selective recognition of integrins. Interestingly, P1 confers enhanced astrocyte targeting when embedded in the AAV9 capsid in respect to AAV6 or AAV2, indicating that the scaffold also plays a crucial role in defining cell tropism (Kunze et al., 2018). Recently, the application of a Cre recombinase-based system enabling the sensitive detection of transgene expression for the *in vivo* selection of a peptide-based AAV9 library allowed the identification of a dominant capsid (AAV-F) whose biodistribution was similar to that of AAV.PHP.B but with higher astrocyte transduction efficiency upon intravenous tail vein injection in C57BL/6 and BALB/c mice (Hanlon et al., 2019b). If the data obtained in rodents translate to large animal models (specifically NHPs) and human models (e.g., hiPSC-derived neural 2D and 3D models), the AAV-F variant could arise as a promising option to less invasively deliver the CRISPR/Cas9 systems into astrocytes. Despite the fact that AAV transduction of microglia is challenging, site-direct mutagenesis of three AAV6 capsid amino acids (Y731F/Y705F/T492V) that prevent proteasomal degradation increased the transduction efficiency of microglia upon ICV injection in P0 pups and after intraparenchymal injection in adult mice (Rosario et al., 2016). The specificity of transgene expression in microglia (75% of total transduced cells) was further increased by using the F4/80 myeloid-specific promoter (Rosario et al., 2016).

Besides the AAV capsid and genome elements, the route of administration and dosing are crucial elements in determining the levels and the homogeneity of biodistribution in CNS regions. Multiple routes of delivery have been evaluated in preclinical models, each showing advantages and disadvantages depending on the particular CNS disease application, targeted tissue or cell type, and the level of transgene expression required to achieve clinical benefits. Even though direct intraparenchymal AAV delivery requires an invasive surgical procedure, preclinical data in rodents and NHPs showed that it is well-tolerated, requires substantially low vector doses to achieve a broad distribution, and displays lower off-target effects in peripheral organs, overall reducing the immunogenicity against the viral particles and transgene (Deverman et al., 2018). This route of administration is particularly relevant to target specific brain regions like white matter areas in demyelinating disorders. The diffusion of the vector can be further increased by the application of the convention-enhanced delivery (CED) system based on a pressure gradient in the infusion catheter leading to expansion of the extracellular space in the brain parenchyma. This leads to a coverage of the brain volume on average 2- to 3-fold higher than classic stereotactic injection techniques (Lonser et al., 2020). Other routes of direct CNS administration include injections into the cerebrospinal fluid (CSF), which should lead to broader AAV distribution. On the one hand, ICV administration is well-tolerated and results in the rostro-caudal coverage of different brain regions. This is particularly true when performed in newborn mice, because the immature ependymal barrier favors the diffusion of the small AAV particles from the CSF into the brain parenchyma (Passini and Wolfe, 2001). While AAV biodistribution was limited in animals injected beyond neonatal day P1, administration in the later periods of post-natal development resulted in an increased non-neuronal transduction with an enhanced rate of astrocyte infection in mice injected at P2 and P3 post-natal days (Chakrabarty et al., 2013). On the other hand, intrathecal administration is particularly relevant in targeting both brain regions and the spinal cord (Bailey et al., 2020; Ballon et al., 2020). The total required AAV dose to achieve widespread AAV biodistribution is generally higher than that injected by the intraparenchymal route but still considerably lower than that used with intravenous administration. Systemic administration has the potential to distribute the AAV particles more uniformly across the entire CNS even when applying non-invasive surgical procedures. Among the different AAV serotypes crossing the BBB, AAV9-PHP.B variants displayed a more than 40-fold higher transduction efficiency of CNS cells when compared with the parental AAV9 after intravenous administration in adult rodents (Deverman et al., 2016; Chan et al., 2017). However, several notable caveats must be considered in the perspective of the clinical translation of systemic AAV administration, including the requirement of higher viral doses and possible undesired off-target delivery to peripheral organs. This could expose the virus to potential antibody neutralization in subjects who have been pre-exposed to natural AAV infections, negatively impacting AAV vector transduction and transgene expression. Additionally, the extent and impact of AAV sequence integration into the host genome are still being debated and might be relevant in the clinical translation of AAV-mediated delivery of CRISPR/Cas9 systems (Hanlon et al., 2019a; Breton et al., 2020).

### Adeno-Associated Vectors to Deliver the CRISPR/Cas9 System to the Central Nervous System

In the context of gene editing, Cas9 expression cassettes containing small-sized nucleases (e.g., SaCas9) under the control of short ubiquitous promoters [e.g., the human elongation factor 1-alpha (EF1-alpha) promoter] allow for the design of an “all-in-one” vector driving simultaneously the expression of sgRNAs. The ICV injection of AAV-SaCas9-sgRNA has been exploited to target the mutant *Sod1* gene. Despite the low (1.52%) InDel frequency at the on-target locus in motor neurons, gene editing resulted in reduced neuroinflammation, ameliorated rotarod performances, and improved life spans of SOD1^G93A^ transgenic mice (Duan et al., 2020). Additionally, an allele-specific editing strategy based on AAV-mediated delivery of the KKH SaCas9 variant has been recently designed to target a point mutation in the *Tmc1* gene, which is responsible for hair cell degeneration and progressive hearing loss in the Beethoven mouse, a model for DFNA36 hearing loss in humans (Gyorgy et al., 2019).

An alternative strategy to deliver large-sized Cas9 orthologs with reduced PAM restrictions is the co-administration of two AAV vectors separately harboring the expression cassettes for the Cas9 nuclease and a sgRNA targeting the desired genomic locus. The advantage of this approach is the possibility to fit cell type-specific promoters into the AAV genome that drive the expression of the Cas9 nuclease, as it was demonstrated by the knock-down of pro-degenerative genes in injured retinal ganglion cells by using the mouse γ-synuclein promoter (Wang et al., 2020a).

Efficient gene correction in neurons and astrocytes via the targeted integration of a donor template has recently been achieved by exploiting the HMEJ pathway based on simultaneous Cas9-mediated cleavages of both the targeted genomic locus and the donor template (containing sgRNA target sites flanking the ~800-bp homology arms). The HMEJ-based method yielded a higher knock-in efficiency in primary astrocytes and neurons when compared with HDR-based strategies and enabled targeted integration in the visual cortex in 50% of transduced cells upon co-delivery of an AAV carrying SpCas9 sequence and a second vector harboring the donor template and sgRNA sequence in adult mice (Yao et al., 2017).

AAV delivery of base and epigenome editors is complicated by the low cargo capacity of the AAV genome. An innovative strategy to overcome this limitation is based on the design of dual intein-split AAV vectors. The first N-intein vector harbors the cytidine or adenine deaminase enzyme fused to the N-terminal portion of nCas9 and is flanked by the N-terminal intein moiety from the *Nostoc punctiforme* (*Npu*). The second N-intein vector carries the C-terminal intein moiety fused to the C-terminal portion of nCas9 in frame with the UGI (only for CBE) and a second expression cassette for the sgRNA (Villiger et al., 2018; Levy et al., 2020; Lim et al., 2020). Split inteins associate post-translationally in a traceless manner, allowing the fusion of the N- and C-terminal portions of nCas9 enzyme in the co-transfected cells and the generation of a fully functional enzyme. Integration of intein-split CBEs and ABEs in optimized vectors (PHP.eB and Anc80) enabled the efficient and robust base editing of DNMT1 upon ICV and retro-orbital injections leading to the correction of *Npc1*^I1061T^ mutation in a mouse model of Niemann–Pick disease type C (Levy et al., 2020).

Epigenome editing in the CNS is an intriguing option to silence mutated genes in autosomal dominant disorders or to activate genes in haplodeficient neurological diseases. Recently, minimal CRISPR activation (CRISPRa) and interference (CRISPRi) transgenes have been generated by fusing catalytically inactive dead SaCas9 to transcriptional activators (VP64 and VP160) or repressors (KRAB and SID4X) along with truncated regulatory elements. A single systemic administration of the PHP.B vector expressing CRISPRa was able to activate the human *SYNAPSIN 1* promoter leading to the expression of a fluorescent reporter transgene in the mouse brain (Lau et al., 2019). In line with these findings, intravenous injections of AAV particles harboring dCas9–VPR and four sgRNAs targeting the upstream promoter region of the voltage-gated sodium channel *Scn1a* gene in Scn1a-haplodeficient mice led to an increased Nav1.1 expression in parvalbumin-positive GABAergic neurons, partially ameliorating the febrile seizures and abnormal behaviors (Yamagata et al., 2020).

Despite these promising preclinical studies on different animal models, several caveats have to be addressed for the perspective clinical translation of AAV-mediated CRISPR/Cas9 delivery to treat neurodegenerative disorders. An estimated 90% of adult humans have been exposed to AAVs, with a substantial fraction of them harboring neutralizing antibodies against the AAV capsid. Humoral and cell-mediated immunity to natural AAV serotypes (Ronzitti et al., 2020) could further exacerbate the pre-existing acquired immune responses against the bacterial Cas nucleases (Gough and Gersbach, 2020; Mehta and Merkel, 2020). Vector design could help in decreasing the immune-mediated toxicities. Molecular engineering of key viral capsid immunogenic amino acids involved in binding with neutralizing antibodies led to the generation of “stealth” AAVs with the ability to avoid pre-existing host immune recognition during gene delivery (Maersch et al., 2010; Smith and Agbandje-McKenna, 2018). AAV-mediated expression of a short hairpin RNA (shRNA) targeting the calcium-dependent scramblase PLSCR1 resulted in reduced vulnerability of transduced cells to microglia clearance as a consequence of the abrogation of the PtdSer externalization that activate TAM-mediated phagocytic clearance (Tufail et al., 2017). Additionally, the use of cell-specific promoters and miRNA de-targeting strategies could restrict the expression to targeted cell populations, thus potentially limiting the Cas9-induced host response.

Integration of the AAV genome occurs at a low frequency in the mammalian genome, but a recent report demonstrated that long-term expression of CRISPR/Cas9 systems substantially impact the AAV integration profile. A high frequency of AAV integration has been observed at the CRISPR cut sites of several genomic loci, including the relevant therapeutic *APP*^SW^ and *Mecp2* genes upon local injection of AAV-Cas9 and AAV-sgRNA vectors in the hippocampus of adult mice (Hanlon et al., 2019a). AAV integration at the on-target locus could negatively impact the efficiency of gene correction strategies based on the integration of donor templates. Additionally, these findings open up several questions on the genotoxicity risk involved with AAV delivery of CRISPR/Cas9 nucleases. Even though the full-length AAV genome is not contained in the majority of integration events, and the genome-wide AAV integration rates outside of the CRISPR on- and off-target loci are not enhanced (Hanlon et al., 2019a), ITR sequences display promoter activity potentially leading to the expression of aberrant transcripts (Earley et al., 2020).

Self-inactivating systems based on the expression of sgRNA knocking down the editing enzymes could potentially reduce the genotoxicity and immune response associated with permanent Cas9 expression (Li et al., 2019). However, these relevant safety issues should be addressed in future preclinical studies before the clinical translation of AAV-based gene editing strategies for the treatment of neurodegenerative disorders.

### Lentiviral Vectors to Deliver Editing Tools to the Central Nervous System

LVs are currently less considered as therapeutic vehicles for conventional gene addition strategies as well as for the delivery of CRISPR/Cas9 systems; however, their peculiar features (i.e., large cargo capacity, negligible viral immunogenicity, and safe integration profile) might overcome some of the limitations associated with AAV vectors in the context of CNS gene addition and gene editing. In patients affected by Parkinson's disease (PD), the 8-year follow-up on ProSavin, an LV delivering key enzymes of the dopamine biosynthetic pathway (tyrosine hydroxylase, aromatic l-amino acid decarboxylase, and guanosine 5′-triphosphate cyclohydrolase 1), documented an improvement of the “off” time in 8/15 treated patients. Additionally, only mild-to-moderate adverse events, absence of tumorigenicity and genotoxicity, and a low and transient immune response against viral particles (detected only in four treated patients) have been reported to demonstrate the safety and tolerability of the therapy (Palfi et al., 2014, 2018).

The higher tropism of LVs for glial cells in rodents and NHPs, including oligodendrocytes (Kagiava et al., 2014; Lattanzi et al., 2014; Ricca et al., 2015; Meneghini et al., 2016), microglia (Wolf et al., 2013; Brawek et al., 2017), and astrocytes (Humbel et al., 2020), defines this delivery system as a good candidate for gene transfer in glial populations (Table 2). The efficacy and safety of LVs for CNS gene therapy have been proven in several preclinical rodent and NHP models of demyelinating diseases, including leukodystrophies (Lattanzi et al., 2010, 2014; Ricca et al., 2015; Meneghini et al., 2016) and multiple sclerosis (Yan et al., 2012; Guglielmetti et al., 2016).

The high cargo capacity (~10 kb) of LV favors the design of an “all-in-one” vector to drive the expression of large size Cas9 nucleases or base editors. The injection of an LV carrying SpCas9 nuclease and a sgRNA targeting the huntingtin coding sequence in the striatum of a mouse model of Huntington disease (HD) resulted in a robust knock-down of the mutant hHTT-82Q protein in both neurons and astrocytes (Merienne et al., 2017). A unique LV-based CRISPR/Cas9 system has recently been generated to simultaneously deliver the Cas9 nuclease and four different sgRNAs, each under the control of a different promoter, thus allowing the simultaneous editing of different cell types in targeted tissues (Kabadi et al., 2014). Additionally, an all-in-one LV carrying dCas9 fused with the catalytic domain of DNA-methyltransferase 3A (DNMT3A) has recently been tested to target *SNCA* triplication in hiPSC-derived dopaminergic neurons to efficiently reduce SNCA expression levels, rescuing mitochondrial ROS production and cellular viability (Kantor et al., 2018; Tagliafierro et al., 2019). Importantly, expression cassettes driven by astrocyte-specific [e.g., gfaABC(1)D and gfaABC(1)D(B3)] and oligodendrocyte-specific [e.g., myelin basic protein (*MBP*) and 2,3-cyclic nucleotide 3-phosphodiesterase (*CNP*)] promoters readily fit within LV genome, favoring glia-specific expression of editing enzymes by using an “all-in-one” system (McIver et al., 2005; Kagiava et al., 2014; Merienne et al., 2017; Humbel et al., 2020).

While LV-based *in vivo* gene addition showed a favorable safety profile, the permanent expression of the editing machinery due to LV genome integration might enhance the immunogenicity against the bacterial Cas9 protein and increase the frequency of off-target and on-target mutagenesis. A self-inactivating system based on the simultaneous expression of an sgRNA targeting a genomic locus (under the strong U6 promoter) and an sgRNA targeting the coding sequence of SpCas9 nuclease (regulated by the weak 7SK promoter) ensured a transient expression of the editing machinery and a low frequency of off-target activity without affecting on-target editing in treated mouse brains (Merienne et al., 2017); however, simultaneous DSBs in the on-target site and within the integrated Cas9 cassette could potentially increase the frequency of deleterious chromosomal translocations. Delivery of CRISPR/Cas9 systems with integration-defective LVs (IDLVs) can overcome this issue, and a recent study showed that IDLV delivery of Cas9 nuclease in the rat ventral striatum resulted in robust gene editing in post-mitotic GABAergic neurons (Ortinski et al., 2017). A potential strategy for transient expression of the CRISPR/Cas9 system is the delivery of an RNA or ribonucleoprotein (RNP) complex in lentiviral capsid-based bionanoparticles. The exploitation of the specific interactions between an aptamer and the aptamer-binding protein (ABP) greatly increased the yield of particles carrying Cas9 mRNA, the cargo RNA copy number, and the genome editing activity (Lu et al., 2019). Additionally, the replacement of the sgRNA scaffold tetraloop with a com aptamer allowed the specific interactions between ABP and the sgRNA within the Cas9 RNP complex and favored its efficient encapsulation in the lentiviral capsid-based bionanoparticles (Lyu et al., 2019). Future *in vivo* studies will assess the efficacy and safety of lentiviral capsid-based bionanoparticles in delivering CRISPR/Cas9 systems.

## Non-viral Methods for Gene Editing Applications

### Nanoparticles to Deliver the CRISPR/Cas9 System to the Central Nervous System

As described above, viral vectors may be very efficient gene-transfer tools, but they still encounter issues when applied for gene transfer of the CRISPR/Cas9 gene editing machinery, including (i) the limited packaging capability, in particular for AAVs; and (ii) the need to tightly regulate Cas9 levels to avoid genotoxic events and immunogenicity due to prolonged Cas9 expression.

For these reasons, non-viral delivery systems have been explored as alternative CRISPR/Cas9 delivery options. In recent years, great advances have been achieved in nanomaterial technologies, leading to improvements in the pharmacokinetic/pharmacodynamic profile and cell-specific delivery of potentially therapeutic molecules. In particular, nanoparticles (NPs) offer several advantages for the efficient delivery of CRISPR/Cas9 RNP complexes. In fact, NPs are tunable in terms of composition, surface functionalization, and degradation rate allowing for (i) increased selectivity for target cells, reducing the risk of potential side effects; (ii) multiple-compound delivery, ranging from small molecules to oligonucleotides and small proteins; and (iii) controlled release over time through modulation of the degradation rate.

The chemical space exploitable to generate NPs is very large due to the fact that it ranges from the use of natural or synthetic polymers to liposomes and micelles, nanogels, or dendrimers. An extensive description of different NP types, their chemical features, and possible drawbacks has been already reviewed (Wei et al., 2020) and will not be covered in this manuscript. Instead, here, we shall summarize successful examples of NP-mediated delivery of CRISPR/Cas9 machinery for *in vivo* applications, highlighting the technical advantages and still unresolved challenges.

Wang et al. (2018b) developed PEGylated NPs based on the PPABLG peptide as an efficient CRISPR/Cas9 delivery system. PPABLG, i.e., poly(γ-4-((2-(piperidin-1-yl)ethyl)aminomethyl)benzyl-l-glutamate), is a α-helical polypeptide that is highly water soluble, carries a cationic side-chain terminus, and is capable of condensing both plasmid DNA and small interfering RNAs (siRNAs). Upon oligonucleotide binding, the polypeptide maintains its helical structure, allowing for membrane penetration and endosomal escape. Thanks to its non-natural amino acid sequence, PPABLG is not recognized by endogenous proteases, thus improving its suitability for *in vivo* applications. PPABLG was used to complex a *Cas9* plasmid and sgRNA (thanks to its highly positive charge) to form nano-complexes, referred by the authors as helical polypeptide NPs (HNPs). The stability of HNPs was enhanced by the incorporation of PEG-Polythymine40 (PEG-T40) in the formulation (referred as P-HNPs). The optimal size of the polyplexes was 100 nm with a z potential (corresponding to the measure of the effective electric charge on the NP surface) of +20. These features were highly dependent on the PPABLG/plasmid DNA ratio. The P-HNPs efficiently delivered the CRISPR/Cas9 system *in vitro* to several cell types, including tumor cells, fibroblasts, dendritic cells, and human endothelial progenitor cells, as well as *in vivo* into tumor cells upon intra-tumoral administration.

Chen et al. (2019) developed a nanocapsule (NC)-mediated CRISPR/Cas9 RNP delivery system. In this case, a mixture of cationic and anionic acrylate monomers was first used to create a coating around the RNPs through electrostatic interactions. The coating was completed by adding imidazole-containing monomers (allowing endosomal escape), a glutathione (GSH)-degradable cross-linker (allowing the release of the RNPs in the cytosol), and acrylate mPEG and acrylate PEG-conjugated ligands (to increase water solubility and allow ligand functionalization, respectively). Finally, an *in situ* free-radical polymerization reaction was initiated to covalently link the monomers, forming an NC around the RNP. Some critical parameters were identified for efficient NC-mediated gene editing: (i) the NC must be biodegradable, to allow the release of RNPs in the cytosol; and (ii) the mass ratios between the NC monomers and RNP affect gene editing efficiencies since NCs with a low NC:RNP ratio (50% of the optimal formulation) were not stable, whereas thicker polymer coating (200% of the optimal formulation) increased the time required to fully degrade the coating and release the RNP. Overall, the average hydrodynamic diameter of the NCs was 25 nm with a relatively neutral zeta potential of −4 mV. *In vivo* studies demonstrated the high efficiency of NC-mediated gene editing upon local injection in the retina or muscles (Chen et al., 2019).

Liu et al. (2020) used bioreducible lipid NPs (LNPs) to deliver Cas9 mRNA and sgRNAs. This represents one of the most efficacious tools for non-viral CRISPR/Cas9 gene editing with an *in vitro* target efficiency of up to ~90% in green fluorescent protein (GFP)-expressing cells, and an *in vivo* editing efficiency of ~80% for the *Pck1* gene, a therapeutic target for cardiovascular disease. The LNPs of this study were composed of disulfide bond-containing hydrophobic tails, based on the lipid BAMEA-O16B. BAMEA-O16B was able to encapsulate mRNA via electrostatic interaction, to assemble NPs, and to allow the release of mRNA intracellularly upon cleavage of the disulfide bonds in response to the reducing environment of the cytoplasm.

Three recent papers reported technical achievements that could open the way for efficient CRISPR/Cas9 delivery to the brain. Lee et al. (2018) demonstrated the rescue of FXS phenotype in *Fmr1* KO mice by targeting *mGluR5* (the metabotropic glutamate receptor isoform 5), which is involved in the exacerbated glutamatergic signaling associated with the FXS pathology. The delivery of spCas9 and Cpf1 RNPs was performed by exploiting the CRISPR-Gold platform. This system is based on gold NPs (15-nm diameter) functionalized with thiol-terminated DNA that allows hybridization with the donor DNA and adsorption of the Cas9 RNP. A layer of silica was then deposited on the NP to increase the negative charge density, and it was then complexed with the cationic endosomal disruptive polymer PAsp(DET). CRISPR-Gold was administered in the striatum of FXS mice, leading to a 40–50% reduction in the expression of the *mGluR5* gene. This was sufficient to significantly ameliorate the behavioral deficits of *Fmr1* KO mice. Interestingly, CRISPR-Gold was found to target astrocytes (33–65% of total target cells, depending on the brain region), microglia (40%), and neurons (3–10%). Park et al. (2019) generated nanocomplexes of 100 nm composed of the Cas9 protein, sgRNA, and the amphiphilic R7L10 peptide. These nanocomplexes were used to target β*-secretase 1* (*Bace1*) gene, obtaining a significant improvement in the cognitive deficits of both 5xFAD and APP knock-in Alzheimer's disease (AD) mouse models. Staahl et al. (2017) performed a direct intracranial injection of uncoated RNPs obtained by condensation of the sgRNA with an SpCas9 containing four copies of the SV40 nuclear localization signal (NLS) at the N-terminal and two copies of the SV40 NLS at the C-terminal (hereafter called 4xNLS–Cas9–2xNLS). The injections were performed in the hippocampus, dorsal striatum, primary somatosensory cortex (S1), and primary visual cortex (V1) of young adult mice. In animals analyzed at 12–14 days post-injection, RNP-mediated gene editing was reported in specific neuronal subtypes, but not in astrocytes or microglia. This suggests that 4xNLS–Cas9–2xNLS RNPs are highly neuron-specific. The molecular mechanisms mediating the neurotropism of 4xNLS–Cas9–2xNLS RNP *in vivo* are not yet understood; however, this approach could pave the way for interesting and promising neuron-specific applications in the context of neurological disease therapy, although the immunogenicity induced by the direct delivery of a bacterial protein still needs to be investigated.

More recently, hyperbranched cationic poly(β-amino ester)s (PBAEs) have gathered an increased interest for gene delivery applications. These materials are composed of pH-sensitive amphiphilic polymers that make PBAEs capable of achieving a robust transfection efficiency, at least *in vitro*, by means of complexation with DNA through electrostatic interaction, and efficient endosomal escape. Rui et al. (2019) recently improved the physicochemical properties of PBAE-based materials by synthesizing a new class of polymers containing both cationic and anionic charges that were end-capped with amino acid-like precursors. This feature made the new compounds capable of complexing proteins (including Cas9 RNPs) in aqueous buffers. These novel PBAEs allowed the delivery of GFP-targeted RNPs in GFP-expressing HEK or GL261 cells, achieving up to 77% and 47% reporter gene KO, respectively. Promising results were also obtained in an orthotopic tumor mouse model, where intra-tumoral infusion of modified PBAEs could achieve efficient RNPs delivery in GL261 cells implanted intracranially.

### Nanoparticle-Mediated Cell-Selective Targeting in the Central Nervous System

Despite the critical advances in RNP formulations strategies, cell specificity remains a big hurdle. To overcome this limitation, the surface functionalization of NPs with cell-specific ligands, antibodies, or well-defined charge profiles could allow preferential internalization in pre-defined cell types. Interestingly, NPs could be modified to contain specific tracers (such as fluorescent dyes, paramagnetic compounds, or radioligands) to allow the tracking of the biodistribution *in vivo* via fluorescent microscopy or through non-invasive approaches such as magnetic resonance imaging (MRI) and positron emission tomography (PET). This is an important aspect, especially for CNS applications, given the difficulty of sampling brain and spinal cord tissues. This may pave the way for more personalized therapeutic approaches for patients, allowing for tuning of the dose and administration protocols of the NPs based on the biodistribution readouts.

Over the last 5 years, several contributions in the field (Elzoghby et al., 2016; Patel and Patel, 2017; Peviani et al., 2019; Birolini et al., 2021) highlighted that the size and surface charge of the NPs are critical features that must be controlled in order to achieve good NP biodistribution in the brain as well as selective cell targeting.

Dimensions and surface charge of NPs critically affect their ability to cross the BBB and to efficiently penetrate/distribute throughout the interconnected multicellular network composing the brain parenchyma. Ideally, the size of NPs should stand in the range of 10–200 nm. In fact, smaller-sized NPs systemically administered are more easily cleared from the circulation by renal filtration; on the other hand, NPs of a relatively large size are not able to permeate across the BBB. Several attempts have tried to overcome these limitations, mainly by exploiting receptor-mediated endocytosis, transcytosis, or transporters. The most widely used method is functionalizing NPs with transferrin, which allows efficient passage through the BBB (Johnsen and Moos, 2016). However, with this strategy, NPs generally remain entrapped in the endothelial cells, which leads to poor delivery to the brain parenchyma. Direct administration of NPs in the CSF, via ICV injection, is an alternative strategy to bypass the BBB. In fact, contrary to the BBB, the ependymal cell layer is more permissive to molecule permeation. In this case, the size and surface charge of the NPs are critical to allow penetration throughout the parenchyma, with smaller-sized and negatively charged NPs achieving the widest biodistribution (Peviani et al., 2019).

Interestingly, there are examples that demonstrate the feasibility of targeting all CNS cell types, including neurons (Dos Santos Rodrigues et al., 2020), oligodendrocytes (Sruthi et al., 2018; Fressinaud et al., 2020), and astrocytes (Vismara et al., 2020). One of the most interesting applications of NPs is the targeting of microglia. Microglial cells, being the macrophagic population of the CNS, possess an intrinsic capacity to efficiently internalize NPs not shielded by PEGylation (Jenkins et al., 2016; Peviani et al., 2019). Indeed, NPs are usually recognized as foreign and uptaken through scavenger receptors, which are highly expressed on microglia (Peviani et al., 2019). Therefore, exploiting microglia-targeted NPs could be a suitable and reliable strategy to deliver genes and/or gene editing machinery to this cell type, which could hardly be amenable to viral vector-mediated targeting.

## Glia Populations as Potential Targets for the Treatment of Neurodegenerative Disorders

Despite the fact that CRISPR/Cas9 technologies have been poorly explored to target astrocytes, oligodendrocytes, and microglia, the genomic editing of glial cells could be an intriguing approach for the treatment of several neurodegenerative disorders. Other than gain-of-function disorders (e.g., AxD), also haploinsufficient disease requiring a fine tune gene regulation is a suitable target for gene editing approaches. The complexity of PLP1 mutations leads to a wide spectrum of pathological phenotypes in PMD patients with the severe form caused by missense PLP1 mutations, intermediate form associated with PLP1/DM20 duplication, and milder form occurring in patients lacking PLP1 expression (Hoffman-Zacharska et al., 2013). Being that gene addition strategies are hard to apply, there is space for the development of editing strategies aimed at correcting PLP1 missense and nonsense mutations or reducing PLP1 expression at physiological levels through the delivery of Cas nucleases, base editors, and epigenome modifiers in OPCs and oligodendrocytes.

Similarly, gene addition strategies based on the ICV administration of a FMR1-AAV9 vector in FMR1-null mice demonstrated that a fine-tuned regulation of FMR1 expression in neurons is required to achieve a therapeutic benefit, being curative only at transgene expression levels between 35 and 115% of physiological levels. Indeed, transgene overexpression had no effect or resulted in neuronal hyperactivity (when ~2.5- to 6-fold WT levels) (Arsenault et al., 2016). The role of astrocytes in the regulation of synaptic connectivity in FXS has been highlighted by *in vitro* co-culture experiments. The co-culture of healthy neurones with astrocytes harboring the FMRP mutation led to an abnormal neuronal dendritic morphology and reduced synaptic connectivity, whereas co-culturing FXS neurones with healthy astrocytes prevented the development of the pathological phenotypes (Jacobs and Doering, 2010; Jacobs et al., 2010). These findings highlight the homeostatic and neuroprotective functions of astrocytes in the FXS neuronal pathology, suggesting that neurons and astrocytes could both be cellular targets for gene editing approaches aimed at restoring physiological FMRP expression (Park et al., 2015; Haenfler et al., 2018; Shitik et al., 2020).

Additionally, changes in the secretome of white matter astrocytes could contribute to demyelination by reducing OPC proliferation and maturation as observed in hiPSC-derived models of Vanishing White Matter (VWM) disease and AxD (Li et al., 2018; Leferink et al., 2019). The release of CH13L1 by astrocytes and its binding to the OPC surface receptor CRTH2 have been suggested to be the main mechanisms impairing OPC proliferation in AxD hiPSC models (Li et al., 2018), making this glycoprotein an interesting target for editing strategies. The CH13L1–CHRT2 pathway as well as other cytokines (Kondo et al., 2016) could be interesting targets to be investigated among different demyelinating disorders.

Reactive astrocytes and reactive microglia have a critical role in several complex and multifactorial neurodegenerative disorders [e.g., AD, amyotrophic lateral sclerosis (ALS), fronto-temporal dementia (FTD), and HD] since they behave as the major drivers of neuroinflammatory responses and influencing disease progression (Bisht et al., 2016; Cai et al., 2017; Pehar et al., 2017; Bright et al., 2019; Gray, 2019; Cipollina et al., 2020; Olah et al., 2020). While transcriptional changes occurring in reactive astrocytes reflect their heterogeneity and show a disease-specific signature (Escartin et al., 2019), the *TREM2* and *APOE* genes were highlighted as two major drivers of the shift toward disease-associated microglia (Krasemann et al., 2017). Interestingly, mutations in these genes have been associated with a poor prognosis and the worst phenotypes of the diseases (Jia et al., 2020; Korvatska et al., 2020; Zhang et al., 2020a). In support of the key role played by TREM2-associated pathways in these diseases, an AAV gene therapy approach targeting the miRNA-mediated downregulation of *CD33* (a gene highly expressed in microglia and acting upstream of the TREM2 pathway) led to a reduction of amyloid beta accumulation and neuroinflammation in an AD mouse model (Griciuc et al., 2020). It would be intriguing to investigate the therapeutic potential of a genome editing approach, allowing for better efficiency of microglia targeting and higher control over the extent of target-gene disruption that could be achieved.

The direct conversion of glial cells in specific neuronal subpopulations or myelinating oligodendrocytes has generated great enthusiasm in the field of regenerative medicine. Decades of *in vitro* and *in vivo* studies have identified several methods to promote transdifferentiation/reprogramming in specific cell types based on bioactive compounds and the ectopic expression of lineage-promoting TFs (Janowska et al., 2019). Neurodegenerative disorders could benefit from the *in vivo* transdifferentiation of astrocytes recruited into the damaged area in dopaminergic neurons (Rivetti di Val Cervo et al., 2017), glutamatergic and GABAergic neurons (Guo et al., 2014; Wu et al., 2020), and interneurons (Su et al., 2014) for the treatment of PD, HD, AD, and spinal cord injuries. Additionally, the possibility to convert *in vivo* resident astrocytes into OPCs or mature oligodendrocytes could be beneficial in restoring the axonal myelination, in particular in focal demyelinating disorders (Mokhtarzadeh Khanghahi et al., 2018; Farhangi et al., 2019). The application of epigenome editors for the *in vivo* transdifferentiation of astrocytes is a novel and intriguing research field. Recently, the restricted expression of SPH (SunTag-p65-HSF1) in the astrocytes of a GFAP-Cre-dependent-SPH transgenic mouse led to the sgRNA-mediated activation of *Ascl1, Neurog2*, and *Neurod1* promoters. This consequently led to the conversion of midbrain astrocytes into functional neurons with a relatively high efficiency (~35%) due to the permanent expression of epigenome editors (Zhou et al., 2018). Further investigation is required to evaluate the efficiency of cell reprograming on the transient expression of epigenome editors, an approach more suitable in the perspective of potential clinical translation for regenerative medicine.

## Conclusions

For a long time, glial cells have been regarded as cells devoted solely to metabolic support and neuronal protection, relegating this cell type to a bystander role in neurodegenerative diseases. This neuron-centric perspective has been challenged over recent years, thanks to the accumulating evidence supporting a central role of glial cells in the maintenance of brain homeostasis, correct development, and physiological functions. The accumulating evidence in patients and animal models highlights the critical involvement of dysfunctional neuron–glia and glia–glia interactions in propelling neuroinflammation and the shift from a neuro-supportive to a neurodegenerative microenvironment. Moreover, in the last few years, several studies have highlighted how the modulation of glial cell reactivity and stimulation of OPC proliferation could help to re-shape a supportive microenvironment and contribute to tissue remodeling, ameliorating the symptomatology of neurodevelopmental, neuropsychiatric, neurodegenerative, and demyelinating disorders (Pinto et al., 2018; Jeon et al., 2020; Kim et al., 2020; Lopez-Guerrero et al., 2020; Miyazaki and Asanuma, 2020; Shippy and Ulland, 2020).

The recent consolidation of advanced editing tools makes the design of strategies for the engineering of glia cells feasible to correct mutations or regulate the expression of disease-causing genes in inherited disorders not amenable to gene addition therapies. Moreover, these technologies could modulate the glial secretome and shift the glial cell reactivity in multi-factorial disorders ranging from AD to demyelinating disorders. The range of potential genomic modifications was expanded by the recent development of prime editing, which employs the conventional CRISPR/Cas systems to mediate all 12 possible base-to-base conversions without conferring DSBs or exploiting a DNA donor template (Anzalone et al., 2019). The LV-mediated delivery of prime editors in murine cortical neurons resulted in the transversion of G–C to T–A nucleotides with an average efficiency of 7.1% in the *Dnmt1* gene, indicating that prime editing is feasible in neural cells (Anzalone et al., 2019).

Gene editing in glia cells is rapidly developing by exploiting technologies previously optimized for gene therapy in the CNS. The design and production of an “all-in-one” or dual vector system, the selection of capsids with higher tropism for specific glia populations, the engineering of promoters with minimal size and cell-specific activity, and the optimization of delivery routes allow the selective expression of editing tools in astrocytes, oligodendrocytes, and microglia. The concomitant development of NP technologies to deliver CRISPR/Cas9 systems for *ex vivo* gene therapy encouraged the first *in vivo* studies targeting the striatum in FXS mice (Lee et al., 2018) and the hippocampus in AD mouse models (Park et al., 2019) with promising results of high on-target editing and low off-target activity.

The safety and efficacy of viral vector platforms in delivering proteins, miRNAs, shRNAs, and long non-coding RNAs in the human brain have been proven in several gene therapy clinical trials (Svetkey et al., 1987; Palfi et al., 2014, 2018; Uchitel et al., 2020). On the contrary, the *in vivo* validation of NP platforms in animal models is still limited, and future studies are required to verify NP stability and diffusion in the brain of large animals (e.g., NHPs). Considering the variety of NP interactions observed in different cell types, *in vitro* assays using human 2D and 3D neural models (e.g., hiPSC-derived neural cultures and brain organoids) could help to understand how efficient the uptake and release of a functional editing complex could be in a human setting.

The extent of target area and cell selectivity could be considered the major determinants in the selection of CRISPR/Cas9 delivery platforms for *in vivo* animal model studies.

The intraparenchymal injection of NPs carrying CRISPR/Cas9 RNAs or RNP is the preferential choice for targeting specific brain regions in focal neurodegenerative disorders, such as AD, PD, and HD. In fact, NP delivery can allow the transient expression of Cas9 nucleases with high on-target editing and low off-target activity, avoiding the genotoxicity associated with viral vector integration at DSB loci and potentially reducing the immune response against the bacterial protein. Although NP-based delivery of base and epigenome editors to various CNS regions must be tested *in vivo*, functionalized lipid-like NPs have been exploited to deliver mRNA encoding an adenine base editor (~5.5 kb) and an sgRNA targeting *Pcsk9* gene in the liver via tail vein injections leading to high levels of A-to-G conversion at the on-target locus (Zhang et al., 2020b).

The major challenge of non-viral delivery is the surface functionalization of NPs with cell-specific ligands, antibodies, or well-defined charge profiles in order to enhance the preferential internalization of CRISPR/Cas9 into astrocytes and oligodendrocytes, reducing the required NP doses injected and avoiding the expression of editors in non-targeted cells. Interestingly, CRISPR-Gold injections in the striatum of FXS mice showed higher targeting of astrocytes compared with microglia and neurons (Lee et al., 2018). Additionally, NP platforms could potentially combine the delivery of editing tools with molecules enhancing HDR pathway (Li et al., 2017; Cao et al., 2020; Maurissen and Woltjen, 2020), decreasing the DSB toxicity (Schiroli et al., 2019), or having therapeutic effects on target cells. Additionally, NPs could be modified to contain specific tracers to track their biodistribution in target tissues via non-invasive MRI or PET techniques. This would allow the direct validation of target engagement and tracking of NP biodistribution in preclinical studies and in clinical settings. Interestingly, the ICV injection of non-PEGylated NPs resulted in efficient internalization in microglial cells, highlighting NPs as a suitable and reliable platform to deliver editing tools that could potentially modulate neuroinflammation in multifactorial disorders.

Treatments of disorders characterized by diffuse neurodegeneration in different regions require NP administration in multiple deposits of the brain parenchyma. On the contrary, a single ICV, intrathecal, or systemic injection of an AAV vector carrying the CRISPR/Cas9 system can ensure a widespread biodistribution of the editing tools. Additionally, the application of self-inactivating systems and miRNA de-targeting strategies that temporally and spatially limit the expression of the CRISPR/Cas9 system could reduce the genotoxicity, cytotoxicity, and immune response triggered by *Cas9* expression. The limited AAV cargo capacity potentially hampers the efficiency of editing strategies based on large size *SpCas9* nucleases, base editors, and epigenome editors whose delivery is based on dual vector systems that require the co-transduction of target cells to be effective. Besides editing efficiency, also safety concerns must be carefully considered, requiring the implementation of a pipeline of preclinical studies with genome-wide analyses, which evaluate the genotoxicity, and immune assays to verify immunogenicity of editing tools before their clinical translation.

**Figure 2:**
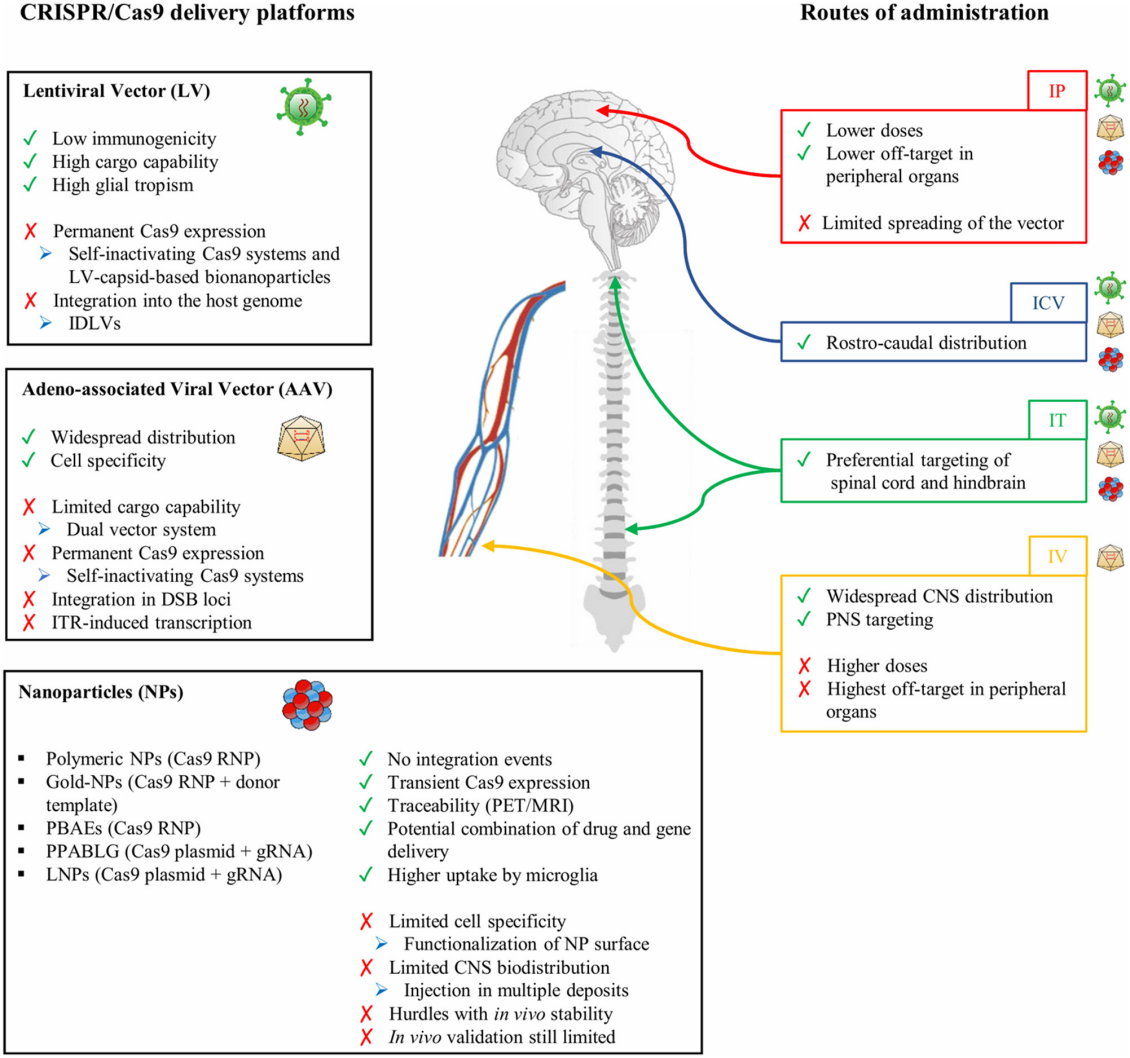
CRISPR/Cas9 delivery platforms and routes of administration. A list of advantages (green) and drawbacks (red) of viral (lentiviral and adeno-associated vectors) and non-viral (nanoparticles) delivery methods. Troubleshooting methods (blue) to overcome potential drawbacks are reported. The delivery routes of viral vectors and nanoparticles are indicated, highlighting their advantages (green) and limitations (red). IP, intraparenchymal; ICV, intracerebroventricular; IT, intrathecal; IV, intravenous; CRISPR, clustered regularly interspaced short palindromic repeats.

The editing of glial cells has great potential to define new strategies for the treatment of both genetic and sporadic neurodegenerative and neurodevelopmental disorders. The modulation of neuron–glia and glia–glia interactions, and the shift of reactive astrocytes and microglia toward an anti-inflammatory and neuroprotective phenotype, could counteract disease progression and favor tissue remodeling in CNS tissues. The delivery of editing tools by viral and non-viral delivery platforms presents different advantages and drawbacks (Figure 2), which depend on the editing strategy, the target cells, and the nature of the disease. Future optimizations in the design of viral vectors and in the formulation of NPs aimed at increasing the editing efficiency and reducing the side effects will favor innovative approaches and their clinical translation to treat neurodegenerative disorders by *in vivo* gene therapy of glia cells.

## Author Contributions

VM, MP, ML, and GZ wrote the paper. VM, MP, and AG revised the manuscript. VM conceived the review. All authors contributed to the article and approved the submitted version.

## Conflict of Interest

The authors declare that the research was conducted in the absence of any commercial or financial relationships that could be construed as a potential conflict of interest.
